# N-myristoyltransferase: A potential novel diagnostic marker for colon cancer

**DOI:** 10.1186/1479-5876-5-58

**Published:** 2007-11-16

**Authors:** Anuraag Shrivastav, Shailly Varma, Anurag Saxena, John DeCoteau, Rajendra K Sharma

**Affiliations:** 1 Department of Pathology and Laboratory Medicine, College of Medicine, University of Saskatchewan, Saskatoon, Canada S7N 5E5; 2 Health Research Division, Saskatchewan Cancer Agency, Saskatoon, Canada S7N 4H4; 3 Department of Biochemistry, College of Medicine, University of Saskatchewan, Saskatoon, Canada S7N 5E5

## Abstract

**Background:**

Colon cancer is the second leading cause of cancer deaths in the western world. If detected early, colorectal cancer is one of the most treatable forms of cancer. Unfortunately, very few people are screened. N-myristoyltransferase (NMT) catalyzes myristoylation of various proteins including oncoproteins. We have demonstrated earlier the alteration of NMT activity during the progression of colorectal cancer and established NMT as a putative therapeutic target for cancer.

**Methods:**

Peripheral blood samples and bone marrow were collected from the colon cancer patients and azoxymethane induced colonic tumor rats and their controls respectively. NMT activity and expression was determined as reported earlier. Immunohistochemical studies were carried out using standard procedures.

**Results:**

In this study we demonstrate for the first time altered expression and localization of NMT in the peripheral blood and bone marrow in colon cancer patients. Immunohistochemical analysis revealed weak to negative staining for NMT in peripheral blood mononuclear cells (PBMC) of controls, whereas strong positivity was observed in PBMC colon cancer patients. In addition, we observed that NMT was localized mostly in the nuclei of the bone marrow (BM) mononuclear cells of the colon cancer patients, whereas NMT remained cytoplasmic in the control bone marrow specimens.

**Conclusion:**

The strikingly different NMT expression offers the basis of a potential adjunct investigative tool for screening or diagnosis of patients at risk for or suspected of having colon cancer. Furthermore, altered localization of NMT in BM of tumor bearing hosts may serve as an added investigative tool for the diagnostic purpose.

## Background

Recently, understanding the lipid modification of proteins has gathered much attention. Protein N-myristoylation is one such lipid modification which involves the covalent attachment of myristate (a 14 carbon saturated fatty acid) to N-terminal glycine of a number of mammalian, viral and fungal proteins [1]. N-myristoylation renders proper function and intracellular trafficking of proteins [2]. Many proteins involved in a wide variety of a signal cascade, cellular transformation and oncogenesis are myristoylated of which most important are several tyrosine kinases such as pp60^src^, pp60^yes^, pp56^lck^, pp59^fyn/syn ^and c-Abl [3]. Myristoylation of proteins is catalyzed by ubiquitously distributed eukaryotic enzyme N-myristoyltransferase (NMT) which is a member of GNAT superfamily of proteins [1-4]. NMT is an emerging therapeutic and drug target [5,6] in various diseases. We have demonstrated earlier the alteration of NMT activity during the progression of colorectal cancer and established NMT as a putative therapeutic target for cancer [7,8].

Colorectal cancer is highly curable if diagnosed early. The primary prognostic approach to identify the differences among the patients with early disease is the Tumor-Node-Metastasis (TNM) system [9]. However, the survival outcome varies among patient with similar pathological disease stage. There have been increasing demands to identify the molecular markers of more aggressive colorectal cancer in order to tailor patient therapy or to identify the disease well in advance.

At present there are several putative biological markers that play important roles in the pathogenesis, proliferation and invasion of colonic tumors. Examples include epidermal growth factor receptor (EGFR), c-MET, β-catenin and p53 [10]. However, a limitation of assessing the expression of these markers or measuring NMT expression and activity for prognostic/diagnostic purposes is that an invasive biopsy must be performed to obtain tumor tissue for protein analysis [8]. Therefore, we have been working towards the detection of NMT in blood samples from colorectal tumor bearing rat and cancer patients.

The result of the present study demonstrates for the first time a simple prognostic/diagnostic tool for colorectal cancer without involving surgery or biopsy. Moreover, this putative marker for colorectal cancer in peripheral blood is not associated with inflammatory response due to the growth of the tumor as is the case for some other markers of colorectal cancer [7].

## Material and methods

Source of chemicals and biochemicals were described earlier [6]. Peptide substrate based on the N-terminal ends of cAMP-dependent protein kinase A (GNAAAKKRR) was synthesized by the Alberta Peptide Institute, Canada. Monoclonal antibodies were purchased from BD Biosciences (Missisuaga, Canada). Polyclonal antibodies was raised against purified human NMT in New Zealand white rabbits and the specificity of this antibody has been described previously [8].

### Blood sample collection

Peripheral blood samples were collected from the colon cancer patients (n = 8) and controls (n = 5) following informed consent according to the guidelines of University of Saskatchewan. Blood smears were prepared for immunohistochemical studies.

### Azoxymethane-induced colonic tumors in rats

All procedures for animal experimentation were carried out in accordance with guidelines of the Canadian Council of Animal Care. Twenty Sprague-Dawley rats (weighing, Males: 250.8 ± 15.5; Female: 168.1 ± 9.02) were obtained from Charles River Canada (St. Constant, Canada). Rats were acclimatized for one week and randomly allocated to treatment or control groups. Twelve rats (six each of male and female) were given eight weekly subcutaneous injections of azoxymetane (10 mg/kg of body weight) in saline. Eight control rats (four each of male and female) were given eight weekly injections of saline only. Animals were given rat chow and rat water ad libitum and housed two per cage. Temperature, humidity and light were controlled at 22°C, 50% and 12/12 hours (light/dark) respectively. Animals were killed after 30 weeks by CO_2 _asphyxiation. Peripheral blood was drawn from tail and blood smears were made on a slide for immunohistochemical studies. Blood from the main artery was collected in vacutainer tubes containing EDTA for the separation of mononuclear cells. The colons were removed followed by flushing of ice-cold saline to remove contents. Further colons were slit longitudinally, and open flat on ice cold surface to count for size and number of tumors.

### Separation of peripheral blood mononuclear cells (PBMC)

Peripheral blood samples were used immediately for the separation of mononuclear cells. PBMC were isolated using Ficoll-Paque according to standard procedures. The isolated PBMC were then resuspended in RPMI medium and cell counts and viability determined. Further, PBMC were lysed in RIPA buffer containing 1 mM PMSF, 10 mM DTT and 1% of protease inhibitor cocktail.

### Isolation of bone marrow

Bone marrow (BM) was obtained from the femurs of rats. BM was obtained from the femoral shafts by flushing it with PBS. Bone marrow was homogenized in RIPA buffer as above. For immunohistochemical studies BM was fixed in 10% formaldehyde and, following dehydration in ascending concentrations of ethanol and xylene, were embedded in paraffin. Five μm thick sections were prepared from archival blocks and placed on glass slides. Bone marrow sections were used from three patients with colon cancer and three controls with no history of any kind of cancer. Bone marrow cells (BMC) were obtained from the femurs of rats. BMC were agitated gently to prepare a single cell suspension and were washed subsequently thrice in PBS. BMC were then incubated for 24 h in a humidified air and 5% CO_2 _at 37°C. After washing the cells remained adhered to the plastic culture plates were bone marrow macrophages. Cells were lysed in RIPA buffer as above and were subjected to Western analysis and NMT assay.

### Immunohistochemistry

Labeled streptavidin-avidin technique was used to localize the primary antibodies after microwave antigen retrieval. The primary antibodies used in this study were: CD3 (polyclonal, 1:80 dilution, Neomarkers, Fairmont, CA), CD20 (monoclonal, 1:20 dilution, Dako, Mississauga, ON), and NMT (polyclonal, 1:50 dilution). Immunohistochemical staining results were evaluated in a semi-quantitative manner as follows: number of mononuclear cells positive: none; rare- 10%; 10–30%, 30–50%, >50%. Staining intensity was evaluated as being absent, weak, moderate or strong.

### N-Myristoyltransferase assay

[^3^H]myristoyl-CoA was synthesized and N-Myristoyltransferase activity was assayed as described previously [6]. The assay mixture contained 40 mM Tris-HCl, pH7.4, 0.5 mM EGTA, 0.45 mM 2-mercaptoethanol, 1% Triton X-100, peptide substrate (500 μM) and NMT in a total volume of 25 μL. The transferase reaction was initiated by the addition of freshly generated [^3^H]myristoyl-CoA and was incubated at 30°C for 30 min. One unit of NMT activity was expressed as 1 pmol of myristoyl peptide formed per min.

### Western blot analysis

SDS-PAGE was carried using standard procedures and Western blot analysis was performed as described previously [6] and probed with monoclonal antibody against NMT-1 (1:250, dilution in blocking buffer). Proteins were estimated by Bradford method using BSA as standard.

## Results and discussion

In thirty weeks all the animals' injected with azoxymethane developed at least two tumors in colon with a maximum of four in two cases. Control rats appeared healthy without any colon tumors. These tumors ranged from adenomas (polyp) to highly invasive tumors.

### N-Myrsitoyltransferase Activity and Expression in Peripheral Blood Mononuclear Cells and Bone Marrow

**Figure 1:**
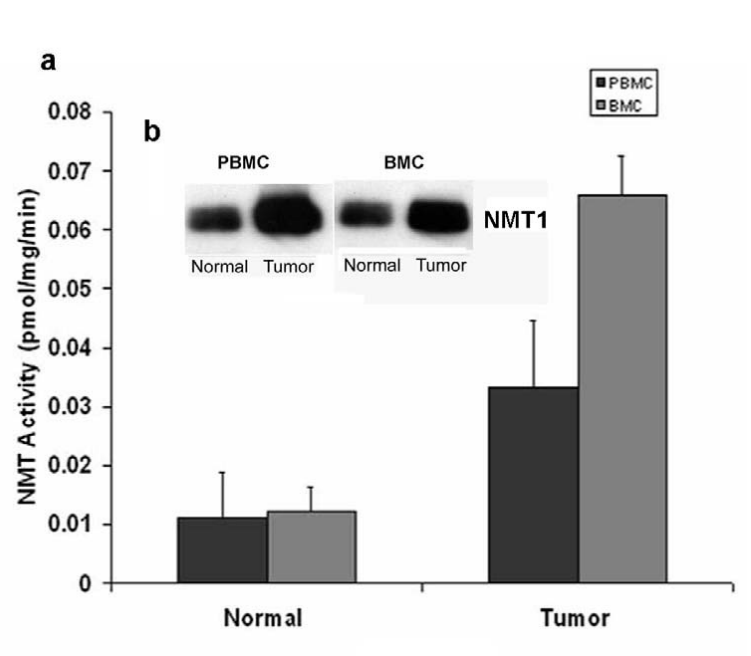
NMT activity in peripheral blood mononuclear cells (PBMC) and Bone Marrow Cells (BMC) of normal and colorectal tumor bearing rats a; Isolated peripheral blood mononuclear cells from peripheral blood of control or tumor bearing rat were assessed for NMT activity. NMT activity was assayed using cAMP-dependent protein kinase derived peptide substrate. Values are mean ± SD of three independent experiments. Inset: Western blot analysis of peripheral blood mononuclear cells and bone marrow cells of normal and colorectal tumor bearing rats. Proteins (25 μg) from PBMC or BMC of control or tumor bearing rats were subjected to 10% SDS-PAGE, transblotted onto nitrocellulose membrane and were probed with monoclonal anti-human NMT antibody (1:250 dilutions).

**Figure 2:**
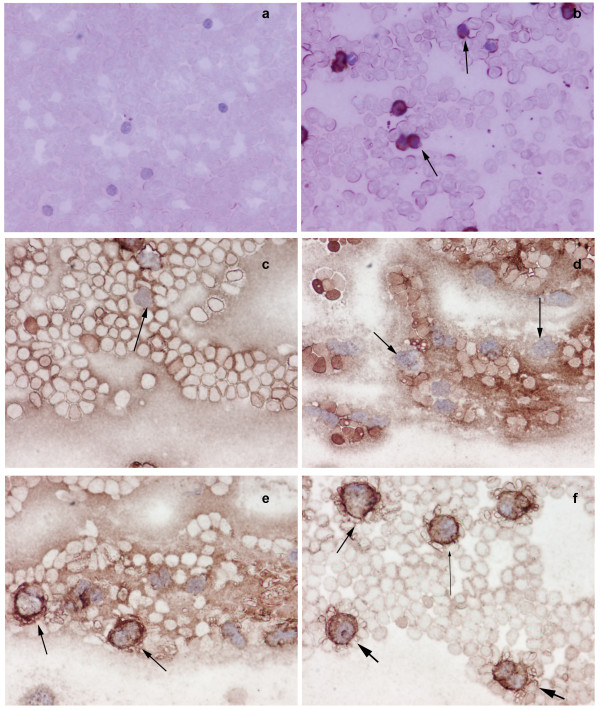
Immunohistochemical analysis of peripheral blood mononuclear cells (PBMC) of normal and tumor bearing hosts. Smears of peripheral blood cells were incubated with anti-NMT antibody. Peripheral blood mononuclear cells (mostly lymphocytes) from control rats were devoid of NMT staining (a), whereas, intense NMT expression was observed in the peripheral blood mononuclear cells of colorectal tumor bearing rats as evident from strong staining (b, see arrows). c negative staining of lymphocytes (see arrow) & d; shows negative staining of monocytes (see arrows) in peripheral blood smear of control e; peripheral blood smear of colon cancer patients show positive staining of macrophages (arrows), f; peripheral blood smear of colon cancer patients show positive staining of neutrophil (fat arrows), lymphocyte (lean long arrow) and macrophages (arrow).

**Figure 3:**
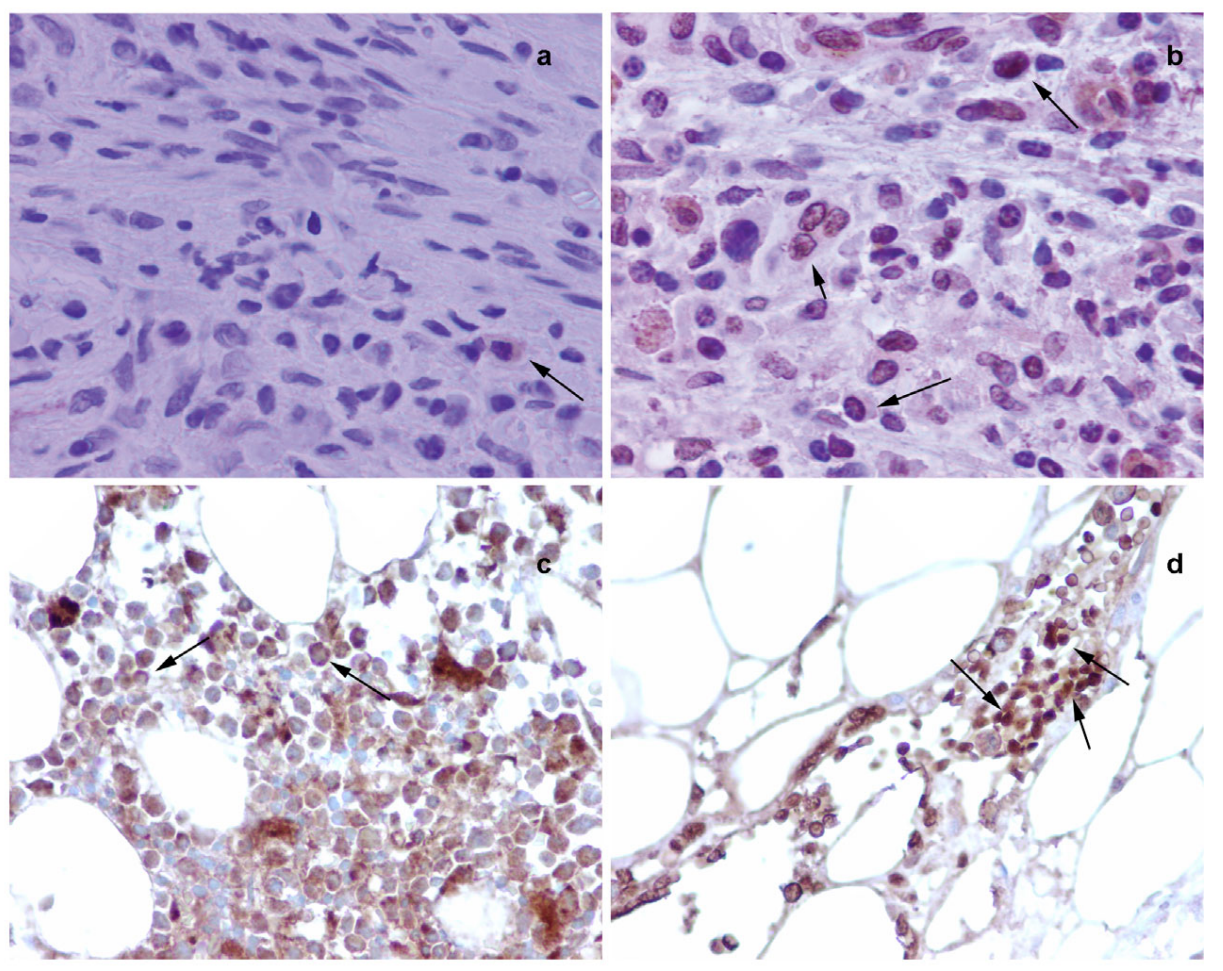
Immunohistochemical analysis of Bone Marrow of normal and tumor bearing hosts. a; shows cytoplasmic staining of NMT (see arrow) in bone marrow cells from control rats, b; whereas, most of the cells shows nuclear localization of NMT in bone marrow cells from tumor bearing rat (see arrow). c; NMT staining is mostly cytoplasmic in bone marrow of control (see arrow). d; Intense nuclear (and some cytoplasmic) staining for NMT is observed in the bone marrow of colon cancer patient (see arrow).

We have established NMT as a putative therapeutic target for colon cancer [7]. To examine that NMT, if at all present in the peripheral blood, can be used as a diagnostic marker for colon cancer, we investigated the expression and activity profile in the peripheral blood of normal or tumor bearing host. NMT activity in the PBMC of tumor bearing rats (n = 20) was higher compared to PBMC of control rats (n = 10). NMT activity was approximately three fold higher in PBMC of tumor bearing rats (Fig. 1). The highest NMT activity, almost 10 fold greater than PBMC of control rats, was observed in the PBMC of two tumor bearing rats with highly invasive tumors. Increased NMT activity in the PBMC may serve as a diagnostic tool for colon cancer. NMT activity was almost five folds higher in bone marrow of tumor bearing rats compared to normal bone marrow (Fig. 1). Highest activity was observed in the bone marrow macrophages of tumor bearing rats. Western analysis was performed to investigate whether higher activity of NMT in PBMC and BM of tumor bearing rats is due to overexpression of NMT or other proteins (activators/inhibitors) are regulating the enzymatic activity. The results revealed that there is overexpression of NMT in the PBMC and BM of tumor bearing rats compared to that of control rats (Fig. 1 inset). Since the activity and expression of NMT in the PBMC is elevated in the colon cancer bearing rats, we further investigated whether the immunohistochemical staining of peripheral blood smear for NMT can be employed for the diagnostic purpose. In rat peripheral blood, the majority of mononuclear cells were CD3+ T-cells with a smaller number of CD20+ (10–15%) B-cells observed. NMT expression was moderate-strong in >50% mononuclear cells in cases with tumors (Fig. 2b) while it was absent or rare weak positivity in controls (Fig. 2a). Intense NMT expression was observed in PBMC of rats with highly invasive tumors. This intense positive staining for NMT in tumor bearing rat tissue indicated its potential use as a diagnostic marker for colon cancer. Therefore, we further extended our study to human patient samples. Patient details are provided in Table 1. In peripheral blood, the majority of the mononuclear cells were CD3 positive T-cells (75 – 80%) admixed with a smaller number of CD20 positive (< 5%) B-cells. CD68 positivity was present in about 15% of cells, mostly monocytes. As can be seen in the Table 1 patient with colon cancer tested positive for NMT in PBMC. Peripheral blood smears from colon cancer patients and healthy controls were stained and probed against anti-CD3, CD68 anti-CD20 and anti-NMT antibodies. NMT staining in the mononuclear cells (including lymphocytes and monocytes) and neutrophils in the peripheral blood smears of the healthy controls ranged from negative to rare weak positivity (Fig. 2c and 2d). Percentage of positive staining for NMT in Control subjects' PBMC is less than 20%. Strong NMT staining was observed in monocytes, lymphocytes and neutrophils in the blood smear of the colon cancer patient (Fig. 2e and 2f). More than 80% cells showed positive for PBMC. In addition, we performed immunohistochemical analysis on the bone marrow sections of colon tumor bearing rats and colon cancer patients. As shown in figure 3b and 3d, NMT was found to be localized in the nuclei as well as in the cytoplasm of the bone marrow mononuclear cells of the tumor bearing rats and colon cancer patients whereas NMT remained cytoplasmic in the control bone marrow specimens (Fig. 3a and 3c). These results indicate that NMT is a potential novel marker for the diagnosis of colon cancer. However, a follow up study with larger samples size is warranted for its validation and development of a blood test for the diagnosis of colon cancer.

**Table 1:** Patient data with NMT positivity for peripheral blood mononuclear cells.

Patient	Age	Sex	Diagnosis	Staging (TNM)	NMT +ve Cells	Intensity
1	74	M	Adenocarcinoma of the traverse colon	pT3_b_, pN0, pMx	> 80%	Strong
2	73	F	Adenocarcinoma of the cecum	pT3a, pN0, pMx	> 80%	Moderate
3	74	F	Adenocarcinoma of the cecum	pT3a, pN0, pMx	30–50%	Moderate
4	66	F	Adenocarcinoma of the ascending colon	pT3, pN0, pM0	30–50%	Weak
5	66	M	Adenocarcinoma of the ascending colon	pT3a, pN0, pMx, pV2	30–50%	Moderate
6	88	M	Adenocarcinoma of the rectum	pT2. pN0, pMx	50–75%	Moderate
7	75	M	Invasive mucinous adenocarcinoma	pT4b, pN2, pMx	> 75%	Moderate
8	83	M	Adenocarcinoma of the rectum	pT3c/d, pN2	> 75%	Strong
Control						
1	45	M			< 5%	Weak
2	34	F			< 5%	Negative
3	23	F			10–15%	Weak
4	59	M			10–15%	Moderate
5	64	M			> 80%	Moderate*

* This was submitted as a control subject and displayed more than 80% positive cells. Later on it was found that the patient had a history of colon cancer.

## Conclusion

The strikingly different NMT expression offers the basis of a potential adjunct investigative tool for screening or diagnosis of patients at risk for or suspected of having colon cancer. Furthermore, altered localization of NMT in BM of tumor bearing hosts may serve as an added investigative tool for the diagnostic purpose. To assess the specificity of the test we performed immunohistochemical staining of the peripheral blood from a chronic lymphocytic leukemic (CLL) patient. Interestingly, NMT staining was negative in the lymphoid cells of the CLL patient (data not shown). A preclinical trial is underway involving larger sample size of patients and healthy controls to validate NMT as blood marker for the diagnosis of colon cancer.

## Competing interests

AS, RKS and University of Saskatchewan currently hold provisional US patent for this work.

## Authors' contributions

All authors have read and approved the final manuscript. A Shrivastav conceived, designed, carried out the studies and drafted the manuscript. SV carried out the studies and drafted the manuscript. A Saxena participated in the immunohistochemical analysis. JD participated in the design of the study. RS conceived the study, and participated in its design and coordination and helped to draft the manuscript. All authors have read and approved the final manuscript.
